# Navigating cancer care in Ukraine: patient’s coping strategies to ensure access and quality

**DOI:** 10.1007/s10552-025-02022-2

**Published:** 2025-09-02

**Authors:** Olena Levenets, Tetiana Chernysh, Milena Pavlova, Wim Groot

**Affiliations:** 1 Department of Health Services Research; CAPHRI, Maastricht University Medical Center, Faculty of Health, Medicine and Life Sciences, Maastricht University, Maastricht, The Netherlands; 2 School of Health Care Management, National University of Kyiv-Mohyla Academy, Kiev, Ukraine; 3 Maastricht Graduate School of Governance, School of Business and Economics, Maastricht University, Maastricht, The Netherlands

**Keywords:** Coping strategies, Informal practices, Informal patient payments, Connections, Ukraine

## Abstract

**Purpose:**

This study investigates how socio-economic and healthcare factors shape the coping strategies of patients diagnosed with cancer in Ukraine, including the decision to forego treatment. The focus is on how these variables influence patients’ strategies to gain access to services, ensure better treatment quality, and decrease treatment costs.

**Methods:**

Data were collected in 2021 through structured interviews in three oncological dispensaries and an online questionnaire among patients diagnosed with cancer in Ukraine (632 patients in total). Sequential logistic regression analyses were applied to identify patterns in selecting specific coping strategies.

**Results:**

The results show that socio-economic characteristics, the perception of service quality, financial resources, and the availability of support influence the choice of coping strategies. Older patients and women are more likely to use informal payments than connections. Patients, who perceive service quality as (very) bad and those finding it hard to afford treatment, are more likely to deploy coping strategies. Higher education and urban residency also impact the coping strategy applied.

**Conclusion:**

The study highlights significant disparities in the choice of coping strategy regarding cancer treatment in Ukraine. Coping strategies, including informal payments and connections, are crucial for accessing and ensuring better treatment. These findings underscore the need for evidence-informed policies to support the most vulnerable cancer care patients in Ukraine.

**Supplementary Information:**

The online version contains supplementary material available at 10.1007/s10552-025-02022-2.

## Introduction

Ukraine is among the 10% of low-, low-middle- and upper-middle-income countries with the lowest government health spending as a share of GDP and high out-of-pocket payments as a share of total health spending [29, 25].

When patients face high out-of-pocket expenditures, many of them resort to coping strategies [39, 40]. In Ukraine, patients diagnosed with cancer rely on connections and informal payments to ensure access to care and to overcome the organizational and financial barriers to treatment [24, 25]. Moreover, out-of-pocket expenditures increase the risk of foregoing treatment [50] and/or facing personal bankruptcy, even in high-income countries [18].

Ukraine also rates poorly on cancer incidence (approximately 160 thousand new cancer cases are registered annually) [11], and an estimated prevalence of over 800 thousand patients living with the disease [11]. However, the full-scale invasion and occupation of Ukrainian regions by Russia and the active internal and external migration of part of the population have negatively affected the quality of quantitative data on morbidity and mortality since 2014 [34]. These data need to be used with some caution and caveats, and they often indicate that the situation is probably worse.

Compared to the EU region, mortality from cancer in Ukraine is slightly higher for people older than 65 and much higher compared to the WHO European region [22]. The mortality-to-incidence ratio (MIR) in Ukraine has been reported to be the highest in Europe, indicating a higher proportion of deaths relative to new cancer cases [12]. This elevated MIR suggests potential gaps in early detection, access to care, and treatment effectiveness. However, over the last 15 years, there has been a slight decrease in the standardized mortality rate by sex in Ukraine [21].

The most common nosological forms of malignant neoplasms morbidity in Ukraine are breast, colorectal and cervical cancer in women, as well as lung, colorectal and gastric cancer in men [34]. The main causes of cancer mortality are breast cancer in women (20% of deaths), and trachea, bronchi and lung cancers in men [21].

According to the national cancer registry of Ukraine, late diagnosis of cancer remains a significant issue, as evidenced by the high percentage of cases detected in the advanced stages of the disease [34]. In 2022, over 38% of cancer cases were identified at 3rd and 4th stages, and 23.9% of patients with cancer died within a year of being diagnosed [34]. Moreover, the working-age population (18–64 years) represented nearly half of the newly diagnosed cases, highlighting opportunities to increase early detection and screening and elevated avoidable cancer mortality [34]. Additionally, the decline in cancer incidence among younger populations, particularly women aged 18–29, may be linked to large-scale migration caused by the war, which disrupted healthcare access and early detection efforts. The persistence of late-stage diagnoses underscores systemic challenges within Ukraine’s healthcare system, including insufficient unsystematic preventive programs and disparities in access to medical care, necessitating policy interventions to enhance early detection and treatment.

In 2020, the National Health Service of Ukraine (NHSU) expanded the national program of medical guarantees to cover all types of care, which resulted in offering 29 specialized care packages, including cancer care [7]. The program of medical guarantees kept expanding, and already in 2021, 35 packages were offered. Each package is contracted separately with health care providers [7]. Despite that, significant gaps in coverage remain, like limited access to modern targeted therapies and immunotherapy. These figures illustrate the relatively low availability of innovative therapies for patients [12]. As a result, the burden of funding due to a lack of treatment and navigating the healthcare system shifts to patients and caregivers and involves an array of coping strategies.

The evidence on coping strategies resulting from barriers to access to cancer treatment (as opposed to studies focused on psychological aspects of coping) is limited. Still, the available studies find that patients use a wide range of coping strategies such as connections, informal payments, and foregoing treatment. These strategies tend to be similar across post-communist countries (countries generally characterized by high organizational barriers to cancer treatment) [26]. However, previous studies have not shed light on important questions, such as how households’ socio-economic status and the financial burden of cancer treatment shape the choice of coping strategies, including the decision to forego treatment [26]. Such evidence is needed to foster evidence-informed policies aimed at patients who are least protected and face the highest risk of poor treatment outcomes.

This study examines how patients diagnosed with cancer choose a coping strategy depending on socio-economic characteristics, the perception of quality of services, financial resources for cancer treatment, and the ability to work. The study also takes into account health conditions, help from non-governmental organizations (NGOs) and the pre-existing network of medical connections/education when trying to gain access to services that are otherwise inaccessible, to ensure better quality of treatment, and to decrease the cost of treatment.

We use data from online and offline surveys conducted in Ukraine in 2021 on various aspects of accessibility, financing, and coping with cancer treatment. We use these data to explore how socio-economic characteristics, the financial burden of cancer treatment, and other relevant factors affect the choice of coping strategies, including the decision not to use any coping strategies when trying to gain access to treatment.

## Methods

Data collection was done in partnership with two NGOs for oncological patients and consisted of two components: structured interviews among one part of the sample and an online questionnaire among the other part of the sample. We surveyed patients who were diagnosed with cancer or who were in remission. For both modes of data collection, the same research instrument (questionnaire) was used.

### Structured interviews

Since there is currently no database with contact information of oncology patients in Ukraine, the sub-sample for the personal structured interviews was formed based on the regional presence of organizations for patients diagnosed with cancer. The sampling was multi-staged: at the first stage, clusters—oncology centers—were selected for collecting data. The survey was conducted in oncology centers in Kharkiv, Odesa, and Kyiv. These large centers represent the Central, Southern, and Eastern parts of Ukraine, and the key representatives of partner oncology patient organizations are located in these cities. During the second stage, all patients (total sampling population) who received medical care at the selected oncology centers during March–September 2021 were invited to participate in the survey and to share their experiences with the treatment they received.

Individual structured interviews were conducted by employees of organizations for patients diagnosed with cancer who were experienced and trained in conducting surveys. All of them participated in a workshop on interviewing given by an employee of the sociological organization Infosapiens.

Altogether, 308 interviews were conducted. In total, 291 respondents in our study were in active treatment, while 17 were not and were excluded from the analysis. Data from 288 interviews were included in the analysis. Also, three questionnaires were disqualified because of incomplete answers.

### Online questionnaire

For the online questionnaire, invitations were distributed among members of patient charities and the Facebook community of oncology patients (10,000 + members) in July–September 2021. The questionnaire was presented on Google form, which was open for 3 months. In total, 574 respondents filled out the online questionnaire. Data from 344 online questionnaires were included in the analysis. The remaining 230 respondents were not in active treatment and were excluded.

### Research instrument

Both online and offline questionnaires were identical and consisted of the following sections: experience of receiving treatment at the hospital, experience with receiving services (surgery, diagnostics, target therapy, hormonal therapy, immunotherapy, radiotherapy, chemotherapy, consultations, drugs), expenditures on treatment and coping with them, and socio-demographic questions. The set of research questions can be found in Appendix 1.

The questionnaire was pre-tested among 10 patients diagnosed with cancer. Data collection during pre-testing was done through both self-completion of the questionnaire and structured interviews.

### Dependent variables

The three dependent variables were included in our regression analyses.

1. A categorical variable indicating which coping strategies were used to gain access to the service that was inaccessible otherwise. The answering categories were: did nothing or didn’t indicate coping strategies; used connections only; used payments only; used patients’ organization information; used only other coping strategies (e.g., demanding and complaining, receiving treatment abroad, finding treatment at a private hospital in Ukraine) and a combination of the following coping strategies: information, payments and connections and other; used any combination of the three dominant coping strategies: information, payments and connections.
2. A categorical variable indicating what coping strategies were used to ensure better quality of the last active treatment. The answering categories were: did nothing or didn’t indicate coping strategies; used connections only; used payments only; used only other coping strategies, e.g., (demanding and complaining, receiving treatment abroad, finding treatment at a private hospital in Ukraine) and a combination of the three coping strategies: information, payments and connections; used any combination of the three dominant coping strategies: information, payments, and connections.
3. A categorical variable indicating what patients did to decrease the cost of treatment. The answering categories were: did nothing; used connections; used payments; used other strategies, namely demanding and complaining, finding cheaper treatment.

### Explanatory variables

There were three groups of explanatory variables in our study (Appendix 2). The first group includes socio-demographic variables: gender, age, education, settlement type, and income. Other studies have shown that women’s risk aversion influences bribery behavior, and that women are more likely to pay informally [19, 32, 41, 42, 52] while Lewis et al., 2000 found that men are more likely to pay [27]. Research also suggests that older people tend to have more limited means for informal payments [10, 20, 35, 28, 45]. There is conflicting evidence regarding the impact of patient’s settlement type and education level [40, 45] on the use of informal payments. Some find that people in rural areas are more willing to use informal payments [5, 10, 20, 28, 48] while others suggest that rural people are less willing to do that [10, 20, 48]. The prevalence of informal payments is higher among people with higher income [10, 19, 29, 32, 42, 48].

The second group includes variables such as the perception of the quality of healthcare services, the difficulty of finding money to pay for treatment, the ability to work, and health status. Although informal practices negatively affect access to quality and affordable healthcare by shifting expenses to out-of-pocket [53], the opposite is also true – informal practices are used to enable access to healthcare services of better quality [30]. The ability to work and poorer health status are also factors that increase the probability of using informal practices [1]. We did not find studies exploring how perceptions of the difficulty of finding money to pay for treatment impact coping strategies, but we believe this variable can affect respondent’s choice of coping strategy given the different options available.

The third group of variables refers to the availability of supportive factors such as information received from NGOs and the availability of medical connections and medical education. Other studies stress the importance of information for patients [47, 16] and the effect of the presence of medical connections or education on the choice of informal practices.

### Data analysis

We applied a sequential regression analysis to identify patterns in the selection of specific coping strategies, their combinations, and aims where these coping strategies were used. In the first stage of the analysis, we compared two groups of respondents: those who used coping strategies versus those who did not use them. In the second stage, we conducted four logistic regressions and compared the group of respondents who used connections with the following groups of respondents who used payments, patients’ organization information, a combination of any other coping strategies (which differs for each dependent variable) and a combination of three dominant coping strategies. Respondents who used connections are the reference category, because connections, if available, are the easiest to use and do not require additional input of resources.

## Results

Altogether, 632 respondents were included through both methods of data collection presented above. Descriptive statistics on the independent variables are presented in Appendix 2. In our dataset, 20% of respondents are male and 80% are female. The average age is 51 years, and 49% of them have master’s or doctorate degrees. In total, 64% live in a city, including Kyiv, and 16% live in a village. In addition, 18% of respondents stated that they have a high income, and 11% stated that they have a very low income. 40% perceived the quality of the last healthcare service as normal, while 18% considered it to be very bad or bad and 11% stated it was very good. For 80% of respondents, it was hard to find money for treatment, and 49% stopped working due to health conditions. In total, 73% do not have medical doctors in their network, while 23% have friends who are medical doctors.

Table in Appendix 3 presents descriptive statistics of the dependent variables. The most commonly used combination of coping strategies included: information, informal payments, connections, looking for information from patients’ organizations, demanding and complaining, receiving treatment abroad, receiving treatment at a private hospital in Ukraine, or using other coping strategies. We found that 32% of respondents used it to gain access to services inaccessible otherwise, and 50% used it to ensure better quality of the last active treatment. When trying to gain access to a service that was inaccessible otherwise, a separate usage of connections or informal payments were the least used strategies by respondents in our study, 9 and 7%, respectively. The least used was a combination of coping strategies (information, informal payments, and connections) when trying to ensure better quality of the last active treatment – only 9% of respondents referred to it. At the same time, 13% of respondents did nothing or did not indicate any coping strategy to gain access to inaccessible services, and 16% did nothing to ensure better quality of the last active treatment.

To decrease the cost of treatment, 45% of respondents used a combination of a few strategies, namely informal payments, connections, demanded and complained, found cheaper service, and foregone some procedures, while 32% did nothing either because they found that the cost of treatment was adequate or because they thought that they could not influence anything. Overall, 17% of respondents in our study used informal payments to decrease the cost of treatment.

Sequential logistic regression analysis was performed for all three dependent variables (see table in Appendix 4). As the first stage in our regression, we analyzed differences between respondents who used coping strategies and those who did not use coping strategies at all. During the second stage, we compared respondents who used connections only to those who used the following coping strategies: only informal payments, patient’s organization information usage, a combination of any other coping strategies, a combination of the three dominant coping strategies, namely information, informal payments, and connections. In the table in Appendix 4, we present results for each independent variable.

The results show that one additional year of age results in a 2.5% decrease in the odds of using all coping strategies vs not using any and 4% higher odds of using informal payments compared to connections when trying to access a service inaccessible otherwise. One additional year of age also results in a 2% increase in the odds of using all strategies vs not using any strategy when trying to ensure better quality of the last treatment. When trying to decrease the cost of treatment, one additional year of age results in 5% higher odds of using a combination of any other strategies vs connections and 4% higher odds of using informal payments compared to connections.

Overall, women have 225% higher odds than men of using coping strategies vs doing nothing when trying to access services inaccessible otherwise. Women have higher odds than men to use payments vs connections when trying to access services inaccessible otherwise and when trying to ensure better quality of the last treatment—352 and 276%—respectively.

Respondents with an incomplete bachelor’s degree, in comparison to respondents with specialized education, have lower odds of using coping strategies vs not using them at all: 58% in case of accessing service inaccessible otherwise; 62% when trying to ensure better quality of the last used service; 35% to decrease the cost of treatment. When it comes to decreasing the cost of treatment, they have 308% higher odds of using other strategies vs connections. Respondents in our study who have master’s or Ph.D. degrees, in comparison to people with specialized education, have 250% higher odds of using a combination of three dominant coping strategies vs using connections only to ensure better quality of the last treatment.

Residents of small cities, in comparison to residents of villages, have 302% higher odds of using coping strategies vs not using them at all, to access services that are otherwise inaccessible and 375% higher odds of using other strategies to decrease the cost of treatment. When it comes to using coping strategies vs not using them at all, residents of Kyiv, in comparison to residents of a village, have higher odds of doing that to access services inaccessible otherwise—209%; to decrease the cost of treatment—225%. When trying to decrease the cost of treatment they have 300% higher odds to use other strategies rather than connections.

Respondents with low, middle, and high income levels, in comparison to respondents with very low income have 76%, 81%, and 87% lower odds correspondingly, of using coping strategies vs not using them all to access services inaccessible otherwise.

Respondents who perceive the quality of services as bad or very bad compared to those who perceived service quality as normal have 275% higher odds of using coping strategies vs not using at all to access services inaccessible otherwise and 362% higher odds of using coping strategies to decrease the cost of treatment and 290% higher odds to use payments vs connections to access services inaccessible otherwise.

Respondents who perceive the quality of services as good in comparison to those who perceive the quality of services as normal have 51% lower odds of using coping strategies to access services inaccessible otherwise and 50% lower odds of using coping strategies to decrease the cost of treatment.

Respondents who perceived the quality of services as very good in comparison to those who perceived the quality of services as normal have 54% lower odds of using coping strategies vs not using and 77% lower odds of using a combination of three dominant coping strategies vs connections and 80% lower odds of using payments vs connections to access services inaccessible otherwise. This group of respondents, when trying to decrease the cost of treatment, has 71% lower odds of using payments vs connections and 89% lower odds of using payments to ensure better quality of the last treatment service.

Respondents for whom it was hard to find the money for treatment, in comparison to those for whom it was not hard, have 210% higher odds of using coping strategies versus not using them at all to access services inaccessible otherwise. They also showed 63% lower odds of using payments vs connections and 67% lower odds of using any other coping strategies vs connections when trying to decrease the cost of the treatment.

Respondents who stopped working due to health conditions, in comparison to those who did not, have 48% higher odds of using all strategies to decrease the cost of treatment and 48% lower odds of using other strategies vs connections when trying to increase quality or the last used service.

Having a doctor in your network in comparison to not having a doctor in your network lowers the odds by 67% of using payments vs connections and by 54% of using other coping strategies vs connections when trying to access services inaccessible otherwise. To ensure better quality of the last treatment, they have 74% times lower odds of using payments vs connections, 49% times lower odds of using other strategies vs connections, and 60% lower odds of using a combination of three dominant coping strategies vs connections.

Respondents who received NGO financial aid in comparison to those who received no help from an NGO have 71% percent lower odds of choosing to use patients’ organization information in comparison to connections when trying to access services that are inaccessible otherwise. Respondents in our study who used NGO informational help have 1000% higher odds of using a combination of all 3 dominant coping strategies vs using connections only to ensure better quality of the last treatment.

## Discussion

According to Lazarus and Folkman, coping is a transaction between the threat, the appraisal, and the response [22]. In this paper, we focus only on problem-based coping strategies, which, according to Lazarus and Folkman [22], are defined as action-based and allow to impact the situation. We not only identify what coping strategies are used as a response and to what threat or goal (e.g., when respondents are trying to access treatment inaccessible otherwise), but we also describe the profile of patients in each case.

The widest possible range of coping strategies combinations, namely using information, informal payments, connections, looking for information from patients’ organizations, demanding and complaining to authorities, receiving treatment abroad, receiving treatment at a private hospital in Ukraine, or using other coping strategies were used by one-third of respondents to gain access to services inaccessible otherwise and by half of the respondents to ensure better quality of the last active service.

As other studies have shown, to sustain treatment-related financial load, patients use lifestyle-altering approaches, and more than 33% use harmful care-altering strategies [34]. Our study complements these findings, showing that to decrease the cost of treatment. Specifically, 45% of respondents used a combination of the following coping strategies: informal payments, connections, demanding and complaining, looking for the cheaper healthcare service, and foregoing some procedures. Interestingly, 17% used informal payments to decrease the cost of treatment. Further research is needed to explore how exactly paying informally reduces the overall cost of treatment. Still, one way is paying informally to the person within healthcare system who is distributing state-provided treatment-related goods/procedures (e.g., chemotherapy, drugs), and can waive the need to purchase it.

While other studies on the impact of age on coping strategies found mixed results and show that problem-focused coping decreases with age [14], or is maintained with age [2], we found that age and gender influence the choice of coping strategy. Older and female respondents have higher odds of using informal payments compared to connections when trying to access services inaccessible otherwise and when trying to decrease the cost of treatment. In line with other studies [43], women more actively use coping strategies. Because of the societal and family roles, they are often the primary person responsible for family health matters. When it comes to explaining the use of informal payments as a coping strategy of primary choice, we see that its monetary nature could be perceived as more binding (toward the recipient), thus a more reliable strategy, so possibly it is used in more important cases such as accessing inaccessible service and decreasing cost of treatment. We also found that older respondents prefer to use a combination of any other strategies over connections when trying to decrease the cost of treatment. This suggests that saving money, or a complete lack of money, might be one of the highest priorities for older patients. Therefore, they deploy a combination of strategies, also due to the fact that with age, the personal network tends to become weaker and less influential [6].

When trying to access services that are inaccessible, the preferred choice for the respondents who perceive the quality of healthcare services as bad or very bad, and for those for whom it was hard to find money for the treatment, is to deploy coping strategies. But then the choice of specific coping strategy differs for these two groups of patients: those who perceive the quality of services as bad or very bad prefer to use payments over connections when trying to access services, while those for whom it was hard to find money for treatment are less likely to use money or any other coping strategies over connections when trying to decrease the cost of treatment.

Respondents with higher education prefer to use a combination of three dominant coping strategies over connections only when trying to ensure better quality of the last treatment, while respondents with higher income are less likely to use coping strategies at all when trying to access services inaccessible otherwise and these odds increase with higher income. Other studies show that a family’s expenditures on healthcare increase with the education level of its members [17, 37, 46] or find an absence of such correlation [38]. The presence of this correlation suggests that educated family members are concerned for the health of their family which drives expenditures up [49]. It is safe to assume that better-educated respondents are using healthcare services more often. Therefore, the range of coping strategies is wider, and they become more flexible and more rational when using available resources. Generally, most studies find that healthcare expenditures increase with income [9, 15, 37]. Therefore, we can argue that instead of choosing to deploy coping strategies, they are more likely to pay out of pocket for the needed healthcare services. In contrast, respondents who stopped working due to health conditions used all strategies to decrease the cost of treatment.

Other studies have shown that inequality in access to healthcare services is caused by both medical supply and socio-economic status, while place of residence is an important factor in both [13, 50, 51]. In our study, we also observe that difference and, hence, the difference in coping strategies. Residents of very big and small cities, in comparison to residents of villages, chose to use coping strategies to access services inaccessible otherwise. When residents of cities aim to decrease the cost of treatment, they use a combination of various coping strategies over connections. This could be explained by the complexity of healthcare services available in the bigger cities and the difficulty navigating these services due to a lack of persistent information in the healthcare system. At the same time, in villages, social connections are usually close [4] and thus may be used as a priority.

We find that respondents with medical knowledge or connections prefer to use connections over any combination of coping strategies when trying to access services inaccessible otherwise or when trying to ensure better quality of the last treatment. This indicates that personal medical connections facilitate navigating healthcare systems and improving access and quality more than affecting cost decisions. Respondents who received informational help from an NGO prefer to use three dominant coping strategies to ensure better quality of the last treatment, while respondents who received financial help from an NGO are less likely to use information from the NGO to gain access to services inaccessible otherwise. This might indicate that NGOs play a critical role in informing patients about alternative access points to healthcare but not in cost reduction.

One of the limitations of the study is that the results of the online survey may not be representative of the country as a whole. However, based on the available representative data, it was possible to compare our data gathered online with the experience of respondents in other regions of the country. Additionally, the combined method of data collection (online and offline questionnaire) allowed us to verify the data collected online. Notably, data in our study were collected before the full-scale Russian invasion of Ukraine which negatively affected access to cancer care, making it even more complicated.

## Conclusion

Results of our study show that patients with cancer in Ukraine resort to a range of coping strategies, including informal payments and leveraging personal connections, to access necessary medical services, ensure better quality of healthcare, and manage the cost of cancer treatment. The reliance on informal practices raises significant concerns about equity in access to healthcare. In our study, patients with fewer resources or weaker social networks may face greater difficulties in accessing treatment, potentially leading to worse health outcomes.

The complex interplay of demographic, socio-economic, and patient’s experiences outlined in our findings underscores the multifaceted challenges faced by patients diagnosed with cancer in Ukraine. In our study, patients from different income levels and regional backgrounds exhibit varying propensities to use coping strategies. Such disparities suggest that coping strategies are not merely a reflection of individual preferences but are heavily influenced by broader socio-economic conditions. Thus, we conclude that policy interventions should consider the socio-economic diversity of patients diagnosed with cancer. Improved affordability and accessibility of cancer care should be the main target of healthcare reforms and once reached, the need for informal practices will subside naturally [3].

## Supplementary Information

Below is the link to the electronic supplementary material.

Supplementary file1 (DOCX 64 KB)

## Data Availability

The datasets generated and analyzed during the current study are not publicly available due to privacy concerns and the sensitive nature of the information collected from participants. However, de-identified data may be made available from the corresponding author upon reasonable request, subject to approval by the institutional ethics committee and in compliance with data protection regulations.
