# Supplementary material for: Navigating cancer care in Ukraine: patient’s coping strategies to ensure access and quality
Source: Cancer Causes Control. 2025 Sep 2;36(12):1775–83. doi: 10.1007/s10552-025-02022-2 (PMC12630248; doi:10.1007/s10552-025-02022-2)
Supplement: Supplementary file 1 — Supplementary file1 (DOCX 64 KB) [file 10552_2025_2022_MOESM1_ESM.docx]

**Appendix 1. Questionnaire**

**A. MEDICAL CARE AT THE ONCOLOGY CLINIC**

A1. Are you currently undergoing active treatment for cancer?

● Yes

● No == proceed to question A10

A2. In which oncology clinic do you receive medical care?

A. Name: ____________________________________________________________

B. City: _____________________________________________________________

A3. Is your active treatment (for this disease) in the form of hospitalization in the oncology clinic or do you come for outpatient services?

● Hospitalization

● Day visits / outpatient treatment

A4. When did you start this course of treatment? _____________ year

A5. How would you rate the quality of services received at the mentioned oncology clinic?

● Very good

● Good

● Average

● Poor

● Very poor

A6. How do you rate the modernity of equipment at this oncology clinic?

● Very good

● Good

● Average

● Poor

● Very poor

A7. How do you rate the attitude of the medical staff at this oncology clinic?

● Very good

● Good

● Average

● Poor

● Very poor

A8. How do you rate the condition of the building (maintenance) inside this oncology clinic?

● Very good

● Good

● Average

● Poor

● Very poor

A9. While at the oncology clinic (both inpatient and outpatient), do you or your relatives have to bring bedding, food:

● Yes

● No

A10. What is your current oncological diagnosis? ____________________________________________

A11. When was this diagnosis established: ___________ year

A12. How do you currently rate your overall health:

● Very good

● Good

● Average

● Poor

● Very poor

A13. How much time during the past four weeks has your physical or emotional health not allowed you to engage in your social activities (such as visiting relatives or friends):

● All the time

● Most of the time

● Some of the time

● A little of the time

● Never

**B. SERVICES**

Surgery

B1.1. Have you undergone surgery related to this diagnosis in the last year?

● Yes

● No == proceed to the next section: Diagnosis

B1.2. How many surgeries have you undergone in the last year? ___ surgeries

B1.3. Where was your last surgery performed?

● Private hospital

● Public hospital

● Both

● Abroad

● Other: _______

B1.4. How much did you or your family pay for the last surgery? _______ UAH

B1.5. How much did you or your family pay unofficially (i.e., gave money directly to medical staff without receiving receipts) for the last surgery? _____ UAH

B1.6. How satisfied are you with how the surgery was conducted?

● Very satisfied

● Satisfied

● Dissatisfied

● Very dissatisfied

**Diagnosis**

B2.1. Have you undergone diagnostic procedures related to this diagnosis in the last year?

● Yes

● No == proceed to the next section: Targeted Therapy

B2.2. How many diagnostic procedures have you undergone in the last year? ___ diagnostics

B2.3. Where was your last diagnostic procedure performed?

● Private hospital

● Public hospital

● Both

● Abroad

● Other: _______

B2.4. How much did you or your family pay for the last diagnostic procedure? _______ UAH

B2.5. How much did you or your family pay unofficially for the diagnostic procedure in the last year? _____ UAH

B2.6. How satisfied are you with how the diagnostic procedure was conducted?

● Very satisfied

● Satisfied

● Dissatisfied

● Very dissatisfied

**Targeted Therapy**

B3.1. Have you undergone targeted therapy related to this diagnosis in the last year?

● Yes

● No == proceed to the next section: Hormonal Therapy

B3.2. How many courses/months of targeted therapy have you undergone in the last year? ___ courses/months of targeted therapy

B3.3. Where was your last targeted therapy performed?

● Private hospital

● Public hospital

● Both

● Abroad

● Other: _______

B3.4. How much did you or your family pay for the last month of targeted therapy? _______ UAH

B3.5. How much did you or your family pay unofficially for targeted therapy in the last year? _____ UAH

B3.6. How satisfied are you with how the targeted therapy was conducted?

● Very satisfied

● Satisfied

● Dissatisfied

● Very dissatisfied

**Hormonal Therapy**

B4.1. Have you undergone hormonal therapy related to this diagnosis in the last year?

● Yes

● No == proceed to the next section: Immunotherapy

B4.2. How many courses/months of hormonal therapy have you undergone in the last year? ___ courses/months of hormonal therapy

B4.3. Where was your last hormonal therapy performed?

● Private hospital

● Public hospital

● Both

● Abroad

● Other: _______

B4.4. How much did you or your family pay for the last month/course of hormonal therapy? _______ UAH

B4.5. How much did you or your family pay unofficially for hormonal therapy in the last year? _____ UAH

B4.6. How satisfied are you with how the hormonal therapy was conducted?

● Very satisfied

● Satisfied

● Dissatisfied

● Very dissatisfied

**Immunotherapy**

B5.1. Have you undergone immunotherapy related to this diagnosis in the last year?

● Yes

● No == proceed to the next section: Radiotherapy

B5.2. How many courses/months of immunotherapy have you undergone in the last year? ___ courses/months of immunotherapy

B5.3. Where was your last immunotherapy performed?

● Private hospital

● Public hospital

● Both

● Abroad

● Other: _______

B5.4. How much did you or your family pay for the last immunotherapy session? _______ UAH

B5.5. How much did you or your family pay unofficially for immunotherapy in the last year? _____ UAH

B5.6. How satisfied are you with how the immunotherapy was conducted?

● Very satisfied

● Satisfied

● Dissatisfied

● Very dissatisfied

**Radiotherapy**

B6.1. Have you undergone radiotherapy related to this diagnosis in the last year?

● Yes

● No == proceed to the next section: Chemotherapy

B6.2. How many radiotherapy sessions have you undergone in the last year? ___ radiotherapy sessions

B6.3. Where was your last radiotherapy performed?

● Private hospital

● Public hospital

● Both

● Abroad

● Other: _______

B6.4. How much did you or your family pay for the last radiotherapy session? _______ UAH

B6.5. How much did you or your family pay unofficially for radiotherapy in the last year? _____ UAH

B6.6. How satisfied are you with how the radiotherapy was conducted?

● Very satisfied

● Satisfied

● Dissatisfied

● Very dissatisfied

**Chemotherapy**

B7.1. Have you undergone chemotherapy related to this diagnosis in the last year?

● Yes

● No == proceed to the next section: Consultations

B7.2. How many chemotherapy sessions have you undergone in the last year? _____ chemotherapy sessions

B7.3. Where was your last chemotherapy performed?

● Private hospital

● Public hospital

● Both

● Abroad

● Other: _______

B7.4. How much did you or your family pay for the last chemotherapy session? _______ UAH

B7.5. How much did you or your family pay unofficially for chemotherapy in the last year? _____ UAH

B7.6. How satisfied are you with how the chemotherapy was conducted?

● Very satisfied

● Satisfied

● Dissatisfied

● Very dissatisfied

**Consultations**

B8.1. Have you received consultations related to this diagnosis in the last year?

● Yes

● No == proceed to the next section: Medications

B8.2. How many consultations related to your diagnosis have you received in the last year? ___ consultations

B8.3. Where was your last consultation conducted?

● Private hospital

● Public hospital

● Both

● Abroad

● Other: _______

B8.4. How much did you or your family pay for the last consultation? _______ UAH

B8.5. How much did you or your family pay unofficially for the consultation in the last year? _____ UAH

B8.6. How satisfied are you with how the consultation went?

● Very satisfied

● Satisfied

● Dissatisfied

● Very dissatisfied

**Medications**

B9.1. Have you taken medications related to this diagnosis in the last year?

● Yes

● No == proceed to the next section: C

B9.2. Where did you last obtain the medications?

● From a doctor in a private hospital

● From a doctor in a public hospital

● From a pharmacy

● From a pharmaceutical representative

● Abroad

● Other: _______

**C. FINANCING TREATMENT AND COPING WITH TREATMENT EXPENDITURES**

C1. How much money did you and your family spend on cancer treatment overall during the past year? _________ UAH.

C2. Were the services you received during treatment [Name only those services from section B that were provided to the patient: surgery/immunotherapy/chemotherapy/diagnosis/targeted therapy/radiotherapy/hormonal therapy] free because they were included in the package of services for oncology patients by the National Health Service of Ukraine (NHSU):

● Yes == proceed to the next question

● No == proceed to question C4

C3. To what extent were the services covered by the National Health Service of Ukraine package [Rate the services listed in section B on a scale from 0 to 10, where 0 means nothing was covered and 10 means everything was covered]?

● Surgery

0 1 2 3 4 5 6 7 8 9 10

● Targeted therapy

0 1 2 3 4 5 6 7 8 9 10

● Chemotherapy

0 1 2 3 4 5 6 7 8 9 10

● Immunotherapy

0 1 2 3 4 5 6 7 8 9 10

● Hormonal therapy

0 1 2 3 4 5 6 7 8 9 10

● Medications

0 1 2 3 4 5 6 7 8 9 10

● Radiotherapy

0 1 2 3 4 5 6 7 8 9 10

● Diagnosis

0 1 2 3 4 5 6 7 8 9 10

● Consultations

0 1 2 3 4 5 6 7 8 9 10

C4. Was it difficult for you and your family to find the money to cover the costs of cancer treatment:

● Not difficult at all == proceed to question C10

● Not too difficult == proceed to question C10

● Normal

● Rather difficult

● Very difficult

Coping Strategies

C5. How many times did you have to forgo the following services [name only those services from section B that were provided to the patient: surgery/immunotherapy/chemotherapy/diagnosis/targeted therapy/radiotherapy/hormonal therapy] during the past year due to lack of funds:

● Surgery ___ times

● Targeted therapy ___ times

● Immunotherapy ___ times

● Hormonal therapy ___ times

● Medications ___ times

● Radiotherapy ___ times

● Diagnosis ___ times

● Consultations ___ times

● Chemotherapy ___ times

C6. What measures did you or your family members take to reduce treatment costs:

● The cost suits me, so I didn't do anything

YES NO

● Did nothing because I don't think I can influence anything

YES NO

● Demanded, complained, appealed to authorities

YES NO

● Used connections

YES NO

● Paid unofficially

YES NO

● Did not use some necessary services

YES NO

● Found a cheaper doctor/service

YES NO

● Other__________________________

C7. Did you or your relatives have to:

● Borrow money == proceed to the next question C8

● Sell property == proceed to question C10

● Friends financially assisted == proceed to question C9

● Seek money from strangers (crowdfunding) == proceed to question C9

● Not applicable == proceed to question C10

C8. How much money did you and your family borrow for treatment during the past year? ________ UAH.

C9. How much money did you and your family receive from friends and collect from strangers during the past year for treatment (besides borrowed)? _________ UAH.

C10. Did you lose your job due to inability to work/health condition:

● Yes

● Partially

● No

C11. Did you receive the following assistance from charitable and patient organizations:

● Did not receive any assistance

YES NO

● Financial

YES NO

● Informational — regarding medical service search, doctor selection, medical center

YES NO

● Informational — regarding free NHSU service packages

YES NO

● Medical recommendations regarding my diagnosis

YES NO

● Psychological support

YES NO

● Items

YES NO

● Medical and non-medical equipment

YES NO

C12. What measures did you or your family take to access a service (during the last active treatment) that would otherwise be unavailable? [Two answer options]

● Used connections

● Paid unofficially

● Sought information through patient communities

● Demanded, complained, appealed to authorities

● Underwent treatment abroad

● Underwent treatment in a private clinic in Ukraine

● The cost suits me, so I did nothing

● Other________

C13. What measures did you or your family take to ensure better quality of the last active treatment? [Two answer options]

● Used connections

● Paid unofficially

● Received treatment abroad

● Received treatment in a private clinic in Ukraine

● The quality suits me, so I did nothing

● Demanded, complained, appealed to authorities

● Consulted a celebrity doctor

● Other________

Personal Connections

C14. Choose two types of services where personal connections were most effective:

● Did not use connections

● Surgery

● Targeted therapy

● Immunotherapy

● Medications

● Radiotherapy

● Chemotherapy

● Diagnosis

● Consultations

C15. For me and my family, it is easy to establish a personal connection with an influential person to achieve our goal during treatment and maintain this connection:

● Completely disagree

● Disagree

● Hard to say

● Agree

● Completely agree

**D. SOCIO-DEMOGRAPHIC CHARACTERISTICS**

E1. Year of birth: _______ year

E2. Your gender:

● Male

● Female

E3. What is your education level:

● Elementary/incomplete secondary

● Complete secondary general

● Vocational/technical

● Incomplete higher/specialized

● Basic higher (bachelor)

● Complete higher (specialist, master)

● Academic degree

E4. Where do you live:

● Capital city

● City (more than 500,000 inhabitants)

● City (200,000 - 500,000 inhabitants)

● Small town up to 200,000

● Village

E5. Which statement best describes your family's financial situation? [One answer]

● We don't have enough money even for food

● We have enough money for food, but buying clothes is difficult

● We have enough money for food and clothes, and we can save some, but not enough to buy expensive items (e.g., TV or refrigerator)

● We can buy some expensive items (e.g., TV or refrigerator), but we cannot make significant savings)

● We can make significant savings

E6. How many people live in your household including yourself: ______ people

E7. Please state the net total income of all members of your household, including all sources of income (wages, social benefits, pensions, rents, fees, etc.) after taxes.?________ UAH.

E8. Do you or any of your relatives/close ones have medical education:

● Yes, I am a doctor

● Yes, there are doctors in my family

● Yes, my friends/acquaintances are doctors

● Hard to say

● No, there are no doctors in my close circle

E9. Have you had experience caring/living with people with cancer diagnoses:

● Yes, I have

● No, I haven't

E10. Your attitude towards the results of healthcare system reform:

● Very good

● Good

● Average

● Bad

● Very bad

● Hard to answer

**Appendix 2. Descriptive statistics of independent variables.**

| **Variable** | Obs. | Mean | Std. Dev. | Min | Max |
| --- | --- | --- | --- | --- | --- |
| **Age: 19 to 82** | 631 | 51.19 | 11.99 | 19 | 82 |
| **Gender** |  |  |  |  |  |
| Gender: male | 632 | 0.20 | 0.40 | 0 | 1 |
| Gender: female | 632 | 0.80 | 0.40 | 0 | 1 |
| **Education** |  |  |  |  |  |
| Education: professional and below | 632 | 0.23 | 0.42 | 0 | 1 |
| Education: incomplete and complete BA | 632 | 0.27 | 0.45 | 0 | 1 |
| Education: complete MA and Doctor | 632 | 0.49 | 0.50 | 0 | 1 |
| **Settlement** |  |  |  |  |  |
| City: village | 632 | 0.16 | 0.37 | 0 | 1 |
| City type: small city | 632 | 0.20 | 0.40 | 0 | 1 |
| City type: city (incl Kyiv) | 632 | 0.64 | 0.48 | 0 | 1 |
| **Income** |  |  |  |  |  |
| Income: very low | 632 | 0.11 | 0.31 | 0 | 1 |
| Income: low | 632 | 0.39 | 0.49 | 0 | 1 |
| Income: middle | 632 | 0.32 | 0.47 | 0 | 1 |
| Income: high | 632 | 0.18 | 0.38 | 0 | 1 |
| **Quality of healthcare services** |  |  |  |  |  |
| Quality: normal | 631 | 0.42 | 0.49 | 0 | 1 |
| Quality of services received:very bad and bad | 632 | 0.18 | 0.38 | 0 | 1 |
| Quality of services received: good | 631 | 0.29 | 0.45 | 0 | 1 |
| Quality of services received: very good | 631 | 0.11 | 0.32 | 0 | 1 |
| **Finding money to pay for treatment** |  |  |  |  |  |
| Not hard to find money | 632 | 0.20 | 0.40 | 0 | 1 |
| Hard to find money | 632 | 0.80 | 0.40 | 0 | 1 |
| **Ability to work and health condition** |  |  |  |  |  |
| Did no stop working due to health condition | 631 | 0.51 | 0.50 | 0 | 1 |
| Stopped working due to health condition | 631 | 0.49 | 0.50 | 0 | 1 |
| **Type of help from NGO** |  |  |  |  |  |
| Did not receive any help | 631 | 0.16 | 0.37 | 0 | 1 |
| Other | 632 | 0.51 | 0.50 | 0 | 1 |
| Financial aid | 632 | 0.11 | 0.31 | 0 | 1 |
| Informational help | 632 | 0.41 | 0.49 | 0 | 1 |
| **Medical education /connections:** |  |  |  |  |  |
| I am not a doctor | 632 | 0.73 | 0.45 | 0 | 1 |
| I am a doctor | 632 | 0.04 | 0.20 | 0 | 1 |
| Friends or relatives are doctors | 632 | 0.23 | 0.42 | 0 | 1 |
| N=632 |  |  |  |  |  |

**Appendix 3. Descriptive statistics of dependent variables included in the analysis.**

| Variable | Obs. | Mean | Std. Dev. |
| --- | --- | --- | --- |
| “What did you do to gain access to the service which was inaccessible otherwise” | | | |
| 0 No coping strategy: only did nothing or didn't indicate coping strategy | 587 | 0.13 | 0.34 |
| 1 use connections only | 587 | 0.07 | 0.26 |
| 2 use informal payments only | 587 | 0.09 | 0.28 |
| 3 use patients’ organization info | 587 | 0.23 | 0.42 |
| 4 used only other strategies and combination of any other coping strategies: information, informal payments, and connections and other | 587 | 0.32 | 0.46 |
| 5 used any combination of the three dominant coping strategies: information, informal payments, and connections | 587 | 0.17 | 0.37 |
| "What did you do to ensure better quality of the last active treatment?" | | | |
| 0 No coping strategy: only did nothing or didn't indicate coping strategy | 584 | 0.16 | 0.37 |
| 1 use connections only | 584 | 0.12 | 0.33 |
| 2 use informal payments only | 584 | 0.13 | 0.34 |
| 3 used only other strategies and used any combination of the three coping strategies: information, informal payments, and connections and other | 584 | 0.50 | 0.50 |
| 4 used any combination of the three dominant coping strategies: information, informal payments, and connections | 584 | 0.09 | 0.28 |
| "What did you do to decrease cost of treatment?" | | | |
| 0 did nothing | 578 | 0.32 | 0.47 |
| 1 used connections | 578 | 0.06 | 0.24 |
| 2 used informal payments | 578 | 0.17 | 0.37 |
| 3 used other strategies | 578 | 0.45 | 0.50 |

**Appendix 4. Results of sequential logistic regression analysis.**

| Variable | **“What did you do to gain access to the service which was inaccessible otherwise?”** | | **"What did you do to ensure better quality of the last active treatment?"** | | **"What did you do to decrease cost of treatment?"** | |
| --- | --- | --- | --- | --- | --- | --- |
|  | base category: did nothing  0 no CS: only did nothing or didn't indicate CS  1 used connections only  2 used payments only  3 used patients’ organization info  4 used only other CS and combination of any other CS: info, payments and connections and other  5 used any combination of the three dominant CS: info, payments and connections | | base category: did nothing  0 no CS: only did nothing or didn't indicate CS  1 used connections only  2 used payments only  3 used only other CS and combination of the three coping strategies: info, payments and connections and other  4 used any combination of the three dominant CS: info, payments, and connections | | base category: did nothing  0 did nothing  1 used connections  2 used payments  3 used other CS | |
|  | OR | Err. | OR | Err. | OR | Err. |
|  | Log likelihood = -845.95148  Number of obs = 584 | | Log likelihood = -723.65416  Number of obs = 582 | | Log likelihood = -630.57909  Number of obs = 575 | |
| **Stage 1:**  **All strategies vs nothing** |  |  |  |  |  |  |
| **Age: 19 to 82** | | | | | | |
|  | 0.97569* | 0.012 | 1.02063* | 0.011 | 0.9984 | 0.009 |
| **Gender: male** | | | | | | |
| Gender: female | 2.25113*** | 0.699 | 1.34972 | 0.421 | 0.97497 | 0.231 |
| **Education: professional and below** | | | | | | |
| Education: incomplete and complete BA | 0.42485** | 0.165 | 0.37607*** | 0.136 | 0.64667* | 0.171 |
| Education: complete MA and Doctor | 0.70055 | 0.274 | 0.63812 | 0.229 | 0.75858 | 0.195 |
| **City: village** | | | | | | |
| City type: small city | 3.02898** | 1.419 | 0.90861 | 0.345 | 1.62837 | 0.495 |
| City type: city (incl Kyiv) | 2.09816** | 0.758 | 1.55083 | 0.527 | 2.25452*** | 0.585 |
| **Income: very low** | | | | | | |
| Income: low | 0.24137** | 0.162 | 1.01558 | 0.462 | 1.15336 | 0.362 |
| Income: middle | 0.19458** | 0.136 | 1.18667 | 0.576 | 1.53261 | 0.524 |
| Income: high | 0.17625** | 0.132 | 1.2562 | 0.684 | 1.21022 | 0.483 |
| **Quality of services received: normal** | | | | | | |
| Quality of services: very bad and bad | 2.75678* | 1.597 | 3.62309** | 1.998 | 1.13055 | 0.31 |
| Quality of services: good | 0.48743** | 0.154 | 0.49821** | 0.135 | 0.82694 | 0.188 |
| Quality of services: very good | 0.46138* | 0.19 | 0.60717 | 0.231 | 0.60829 | 0.199 |
| **Find money for treatment: not hard** | | | | | | |
| Find money for treatment: hard | 2.10428** | 0.677 | 1.31108 | 0.389 | 0.68886 | 0.189 |
| **Stopped working due to a health condition: no** | | | | | | |
| Stopped working due to a health condition: yes | 1.55986 | 0.463 | 1.4983 | 0.384 | 1.48289** | 0.291 |
| **NGO: did not receive any help** | | | | | | |
| NGO: received financial aid | 1.04358 | 0.743 | 0.86431 | 0.422 | 1.47192 | 0.629 |
| NGO: information help | 2.33309 | 1.676 | 1.3234 | 0.708 | 1.89822 | 0.874 |
| NGO: other | 0.84938 | 0.616 | 1.47034 | 0.831 | 0.94045 | 0.443 |
| **I am a doctor: no** | | | | | | |
| I am a doctor: yes | 0.71236 | 0.442 | 1.00763 | 0.594 |  |  |
| Friends or relatives are doctors | 1.09146 | 0.375 | 1.07304 | 0.315 |  |  |
| **Stage2:**  **Payments vs connections** | | | | | | |
| **Age: 19 to 82** | | | | | | |
|  | 1.04059* | 0.021 | 1.02071 | 0.016 | 1.03902** | 0.02 |
| **Gender: male** | | | | | | |
| Gender: female | 3.52273* | 2.369 | 2.76916* | 1.532 | 1.54835 | 0.873 |
| **Education: professional and below** | | | | | | |
| Education: incomplete and complete BA | 0.37001 | 0.24 | 0.4429 | 0.221 | 1.23049 | 0.786 |
| Education: complete MA and Doctor | 0.85168 | 0.532 | 1.01579 | 0.454 | 0.59288 | 0.314 |
| **City: village** | | | | | | |
| City type: small city | 3.72048 | 3.242 | 0.76801 | 0.469 | 2.63776 | 1.891 |
| City type: city (incl Kyiv) | 2.14966 | 1.668 | 0.64021 | 0.324 | 2.20296 | 1.27 |
| **Income: very low** | | | | | | |
| Income: low | 3.09255 | 2.361 | 1.60545 | 0.967 | 0.38804 | 0.347 |
| Income: middle | 1.53819 | 1.238 | 1.44052 | 0.907 | 0.66288 | 0.611 |
| Income: high | 0.99157 | 0.966 | 1.01121 | 0.754 | 0.31248 | 0.316 |
| **Quality of services received: normal** | | | | | | |
| Quality of services: very bad and bad | 2.90069* | 1.805 | 1.6684 | 0.736 | 0.91561 | 0.567 |
| Quality of services: good | 0.93791 | 0.536 | 0.94802 | 0.425 | 0.58246 | 0.309 |
| Quality of services: very good | 0.20802* | 0.188 | 0.11680* | 0.13 | 0.09335*** | 0.069 |
| **Find money for treatment: not hard** |  |  |  |  |  |  |
| Find money for treatment: hard | 1.71739 | 1.17 | 0.81273 | 0.405 | 0.27740* | 0.184 |
| **Stopped working due to a health condition: no** | | | | | | |
| Stopped working due to a health condition: yes | 1.06747 | 0.504 | 1.12515 | 0.423 | 0.73724 | 0.327 |
| **NGO: did not receive any help** | | | | | | |
| NGO: received financial aid | 0.24905 | 0.26 | 0.20753 | 0.204 | 1.42405 | 1.156 |
| NGO: information help | 0.23914 | 0.25 | 0.57494 | 0.527 | 0.4951 | 0.515 |
| NGO: other | 0.59833 | 0.646 | 0.40425 | 0.378 | 0.58637 | 0.64 |
| **I am a doctor: no** | | | | | | |
| I am a doctor: yes | 1.23661 | 1.848 | 1.71995 | 2.208 |  |  |
| Friends or relatives are doctors | 0.33315** | 0.176 | 0.26189*** | 0.109 |  |  |
| **Patient organization information vs connections** | | | | | | |
| **Age: 19 to 82** | | | | | | |
|  | 1.0023 | 0.017 |  |  |  |  |
| **Gender: male** | | | | | | |
| Gender: female | 1.68066 | 0.884 |  |  |  |  |
| **Education: professional and below** | | | | | | |
| Education: incomplete and complete BA | 0.55486 | 0.314 |  |  |  |  |
| Education: complete MA and Doctor | 0.69589 | 0.389 |  |  |  |  |
| **City: village** | | | | | | |
| City type: small city | 1.20833 | 0.829 |  |  |  |  |
| City type: city (incl Kyiv) | 0.50141 | 0.299 |  |  |  |  |
| **Income: very low** | | | | | | |
| Income: low | 1.97014 | 1.34 |  |  |  |  |
| Income: middle | 2.00512 | 1.394 |  |  |  |  |
| Income: high | 2.23059 | 1.78 |  |  |  |  |
| **Quality of services received: normal** | | | | | | |
| Quality of services: very bad and bad | 2.07587 | 1.142 |  |  |  |  |
| Quality of services: good | 1.40931 | 0.662 |  |  |  |  |
| Quality of services: very good | 0.70076 | 0.398 |  |  |  |  |
| **Find money for treatment: not hard** | | | | | | |
| Find money for treatment: hard | 1.15423 | 0.587 |  |  |  |  |
| **Stopped working due to a health condition: no** | | | | | | |
| Stopped working due to a health condition: yes | 1.33832 | 0.528 |  |  |  |  |
| **NGO: did not receive any help** | | | | | | |
| NGO: received financial aid | 0.28425* | 0.206 |  |  |  |  |
| NGO: information help | 0.81344 | 0.666 |  |  |  |  |
| NGO: other | 0.25885 | 0.228 |  |  |  |  |
| **I am a doctor: no** | | | | | | |
| I am a doctor: yes | 2.63248 | 2.985 |  |  |  |  |
| Friends or relatives are doctors | 0.56126 | 0.228 |  |  |  |  |
| **Combination of any other coping strategies vs connections** | | | |  |  |  |
| **Age: 19 to 82** | | | | | | |
|  | 1.01453 | 0.017 | 1.01215 | 0.012 | 1.04550** | 0.019 |
| **Gender: male** | | | | | | |
| Gender: female | 1.02602 | 0.493 | 0.70335 | 0.271 | 1.0397 | 0.521 |
| **Education: professional and below** | | | | | | |
| Education: incomplete and complete BA | 0.4109 | 0.222 | 1.15194 | 0.45 | 3.07393* | 1.849 |
| Education: complete MA and Doctor | 0.64668 | 0.345 | 1.48377 | 0.558 | 1.28243 | 0.632 |
| **City: village** | | | | | | |
| City type: small city | 0.79077 | 0.544 | 0.86739 | 0.437 | 3.75115** | 2.504 |
| City type: city (incl Kyiv) | 1.1606 | 0.671 | 0.98984 | 0.421 | 3.00173** | 1.617 |
| **Income: very low** | | | | | | |
| Income: low | 1.20754 | 0.749 | 1.41704 | 0.701 | 0.33599 | 0.285 |
| Income: middle | 0.58679 | 0.379 | 0.83676 | 0.431 | 0.42003 | 0.369 |
| Income: high | 0.71387 | 0.534 | 1.07228 | 0.635 | 0.24855 | 0.234 |
| **Quality of services received: normal** | | | | | | |
| Quality of services: very bad and bad | 1.26262 | 0.676 | 0.97769 | 0.366 | 1.8808 | 1.072 |
| Quality of services: good | 0.77947 | 0.357 | 1.2281 | 0.436 | 1.37289 | 0.666 |
| Quality of services: very good | 0.56172 | 0.296 | 1.17529 | 0.549 | 0.29444** | 0.175 |
| **Find money for treatment: not hard** | | | | | | |
| Find money for treatment: hard | 1.08177 | 0.53 | 0.99528 | 0.384 | 0.33138* | 0.205 |
| **Stopped working due to a health condition: no** | | | | | | |
| Stopped working due to a health condition: yes | 0.66877 | 0.255 | 0.52233** | 0.156 | 0.89537 | 0.364 |
| **NGO: did not receive any help** |  |  |  |  |  |  |
| NGO: received financial aid | 0.58126 | 0.412 | 1.02401 | 0.686 | 0.54656 | 0.413 |
| NGO: information help | 0.60384 | 0.479 | 0.69065 | 0.494 | 0.65519 | 0.65 |
| NGO: other | 0.69776 | 0.592 | 0.45577 | 0.339 | 0.2815 | 0.291 |
| **I am a doctor: no** |  |  |  |  |  |  |
| I am a doctor: yes | 1.69628 | 1.904 | 2.56208 | 2.742 |  |  |
| Friends or relatives are doctors | 0.46294* | 0.184 | 0.51567** | 0.155 |  |  |
| **Combination of the three dominant coping strategies vs connections** | | | | |  |  |
| **Age: 19 to 82** | | | | | | |
|  | 1.00056 | 0.018 | 0.99317 | 0.018 |  |  |
| **Gender: male** | | | | | | |
| Gender: female | 1.1986 | 0.649 | 1.19747 | 0.701 |  |  |
| **Education: professional and below** | | | | | | |
| Education: incomplete and complete BA | 0.76224 | 0.466 | 1.00799 | 0.603 |  |  |
| Education: complete MA and Doctor | 1.57398 | 0.937 | 2.50515* | 1.374 |  |  |
| **City: village** | | | | | | |
| City type: small city | 1.29622 | 0.923 | 1.01377 | 0.716 |  |  |
| City type: city (incl Kyiv) | 0.61603 | 0.385 | 0.94785 | 0.576 |  |  |
| **Income: very low** | | | | | | |
| Income: low | 3.0886 | 2.282 | 3.27626 | 2.622 |  |  |
| Income: middle | 2.42026 | 1.84 | 2.28721 | 1.907 |  |  |
| Income: high | 1.5422 | 1.352 | 1.03005 | 0.99 |  |  |
| **Quality of services received: normal** | | | | | | |
| Quality of services: very bad and bad | 1.11449 | 0.654 | 0.72882 | 0.395 |  |  |
| Quality of services: good | 1.39298 | 0.666 | 1.07451 | 0.52 |  |  |
| Quality of services: very good | 0.23230** | 0.165 | 0.71096 | 0.502 |  |  |
| **Find money for treatment: not hard** | | | | | | |
| Find money for treatment: hard | 1.08534 | 0.584 | 1.02439 | 0.569 |  |  |
| **Stopped working due to a health condition: no** | | | | | | |
| Stopped working due to a health condition: yes | 1.58901 | 0.657 | 1.26772 | 0.53 |  |  |
| **NGO: did not receive any help** | | | | | | |
| NGO: received financial aid | 0.6787 | 0.501 | 3.05767 | 2.541 |  |  |
| NGO: information help | 1.72721 | 1.557 | 10.13648* | 13.278 |  |  |
| NGO: other | 0.87474 | 0.847 | 8.41592 | 11.459 |  |  |
| **I am a doctor: no** | | | | | | |
| I am a doctor: yes | 2.01189 | 2.405 | 1.22793 | 1.812 |  |  |
| Friends or relatives are doctors | 0.54517 | 0.234 | 0.40726** | 0.182 |  |  |
| Standard errors in parentheses,  * p<0.10, ** p<0.05, *** p<0.01 |  |  |  |  |  |  |

**References**

1. Amiri, M. M., Bahadori, M., Motaghed, Z., & Ravangard, R. (2019). Factors affecting informal patient payments: a systematic literature review. International Journal of Health Governance, 24(2), 117-132.

1. Aldwin, C. M. (1991). Does age affect the stress and coping process? Implications of age differences in perceived control. The Journals of Gerontology: Series B, 46(6), P174–P180.
2. Baji P., Pavlova M., Gulácsi L., Farkas M., Groot W. (2014). The link between past informal payments and willingness of the Hungarian population to pay formal fees for health care services: results from a contingent valuation study. Eur J Health Econ, 15(8), 853–867. <https://doi.org/10.1007/s10198-013-0531-y>
3. Business Group on Health. [https://www.businessgrouphealth.org/en/resources/voluntary%20health%20insurance%20landscape%20in%20ukraine](https://www.businessgrouphealth.org/en/resources/voluntary%252520health%252520insurance%252520landscape%252520in%252520ukraine)
4. Cantarero D., Lago-Peñas S. (2010). The determinants of health care expenditure: a reexamination. Appl Econ Lett, 17(7), 723–726. <https://doi.org/10.1080/13504850802314437>
5. Cockcroft A., Andersson N., Paredes-Solis S., Caldwell D., Mitchell S., Milne D., et al. (2008). An inter-country comparison of unofficial payments: results of a health sector social audit in the Baltic States. BMC Health Serv Res, 8(1), 15. <https://doi.org/10.1186/1472-6963-8-15>
6. "Cancer in Ukraine 2020-2021: incidence, mortality, prevalence and other relevant statistics. Bulletin of the National Cancer Registry of Ukraine. Vol. 23". http://www.ncru.inf.ua/publications/BULL_23/index_e.htm. Accessed January 1, 2022.
7. Fleurbaey M., Schokkaert E. (2011). Equity in health and health care. Handbook Health Econ, 2, 1003–1092. <https://doi.org/10.1016/B978-0-444-53592-4.00016-5>
8. Folkman S., Lazarus RS, Pimley S, Novacek J. (1987). Age differences in stress and coping processes. Psychol Aging, 2, 171–184. <https://doi.org/10.1037/0882-7974.2.2.171>
9. Furuoka F., Lim BFY, Kok E, et al. (2011). What are the determinants of health care expenditure? Empirical results from Asian countries. Sunway Acad J, 8, 12–25.
10. Hartshorne J., Carstens I. (1990). Role of information systems in public health services.
11. Hatam N., Tourani S., Rad EH, et al. (n.d.). Estimating the relationship between economic growth and health expenditure in ECO countries using panel cointegration.
12. Himmelstein D. U., Thorne D., Warren E., Woolhandler S. (2009). Medical bankruptcy in the United States, 2007: results of a national study. The American journal of medicine, 122(8), 741-746. <https://doi.org/10.1016/j.amjmed.2009.04.012>
13. Kankeu HT, Boyer S, Fodjo Toukam R, Abu-Zaineh M. (2016). How do supply-side factors influence informal payments for healthcare? The case of HIV patients in Cameroon. Int J Health Plann Manag, 31(1), 41–57. <https://doi.org/10.1002/hpm.2266>
14. Khodamoradi A., Rashidian A., Aghlmand S., Arab M. (2015). Informal payments and its related factors in Urmia hospitals. Hakim Res J, 17(4), 313–321.
15. Lazarus, R. S. (1984). Stress, appraisal, and coping (Vol. 464). Springer.
16. Lekhan, V. N., Zaiarskyi, M. I., Vudvud, V. V., Kovalevych, D. A. (2022). National health expenditure trends, 2000 to 2019. Wiadomości Lekarskie, 75(5 p 1), 1141-1147.
17. Levenets, O., Stepurko, T., Pavlova, M., Groot, W. (2019a). Coping mechanisms of Ukrainian patients: bribes, gifts, donations, and connections. In Governance beyond the Law (pp. 125-143). Palgrave Macmillan, Cham.
18. Levenets, O., Stepurko, T., Polese, A., Pavlova, M., Groot, W. (2019b). Coping strategies of cancer patients in Ukraine. The International journal of health planning and management, 34(4), 1423-1438.
19. Levenets, O., Stepurko, T., Polese, A., Pavlova, M., Groot, W. (2021). Coping with cancer in post-communist Europe: a systematic literature review. Health Policy and Planning, 36(10), 1690-1704.
20. Lewis M. (2000). Who is paying for healthcare in Eastern Europe and Central Asia?. World Bank Publications.
21. Lewis M. (2007). Informal payments and the financing of health care in developing and transition countries. Health Aff, 26(4), 984–997. <https://doi.org/10.1377/hlthaff.26.4.984>
22. Liaropoulos L., Siskou O., Kaitelidou D., Theodorou M., Katostaras T. (2008). Informal payments in public hospitals in Greece. Health Policy, 87(1), 72–81. <https://doi.org/10.1016/j.healthpol.2007.12.005>
23. Mæstad, O., Mwisongo, A. (2007, June). Informal payments and the quality of health care in Tanzania: results from qualitative research. CMI Working Paper, iHEA 2007 6th World Congress: Explorations in Health Economics Paper.
24. Meskarpour Amiri, M., Teymourzadeh, E., Ravangard, R., Bahadori, M. (2019). Health informal payments and their main determinants: the case of Iran. Proceedings of Singapore Healthcare, 2010105818822594. <https://doi.org/10.1177/2010105818822594>
25. Miller WL, Grødeland ÅB, Koshechkina TY. (2000). If you pay, we'll operate immediately. J Med Ethics, 26(5), 305–311. <https://doi.org/10.1136/jme.26.5.305>
26. Nipp, R. D., Zullig, L. L., Samsa, G., Peppercorn, J. M., Schrag, D., Taylor Jr, D. H., ... & Zafar, S. Y. (2016). Identifying cancer patients who alter care or lifestyle due to treatment‐related financial distress. Psycho‐Oncology, 25(6), 719-725.
27. Piroozi B., Rashidian A., Moradi G., Takian A., Ghasri H., Ghadimi T. (2017). Out-of-pocket and informal payment before and after the health transformation plan in Iran: evidence from hospitals located in Kurdistan, Iran. Int J Health Policy Manag, 6(10), 573. <https://doi.org/10.15171/ijhpm.2017.16>
28. Pourtaleb, A., Jafari, M., Seyedin, H., Akhavan Behbahani, A. (2020). New insight into the informal patients’ payments on the evidence of literature: a systematic review study. BMC Health Services Research, 20, 1-11. <https://doi.org/10.1186/s12913-019-4647-3>
29. Rahman T. (2008). Determinants of public health expenditure: some evidence from Indian States. Appl Econ Lett, 15(11), 853–857. <https://doi.org/10.1080/13504850600770970>
30. Sekhar RH. (2006). Influence of income and education on household health expenditure: the case of tribal Orissa. The Orissa J Commer, 28, 133–144.
31. Shi, L., Starfield, B., Kennedy, B., Kawachi, I. (1999). Income inequality, primary care, and health indicators. J Fam Pract, 48(4), 275-284.
32. Starfield, B., Gérvas, J., & Mangin, D. (2012). Clinical care and health disparities. Annual review of public health, 33(1), 89-106. <https://doi.org/10.1146/annurev-publhealth-031811-124528>
33. Stepurko T, Pavlova M, Gryga I, Groot W. (2013). Informal patient payments in maternity hospitals in Kiev, Ukraine. Int J Health Planning Manag, 28(2), 169–187. <https://doi.org/10.1002/hpm.2155>
34. Stepurko T, Pavlova M, Gryga I, Groot W. (2015). To pay or not to pay? A multicountry study on informal payments for healthcare services and consumers’ perceptions. Health Expect, 18(6), 2978–2993. <https://doi.org/10.1111/hex.12281>
35. Tamres, L. K., Janicki, D., Helgeson, V. S. (2002). Sex Differences in Coping Behavior: A Meta-Analytic Review and an Examination of Relative Coping. Personality and Social Psychology Review, 6(1), 2-30. <https://doi.org/10.1207/S15327957PSPR0601_1>
36. Tambor, M., Pavlova, M., Rechel, B., Golinowska, S., Sowada, C., Groot, W. (2014). The inability to pay for health services in Central and Eastern Europe: evidence from six countries. European journal of public health, 24(3), 378–385. <https://doi.org/10.1093/eurpub/ckt118>
37. Tomini S., Maarse H. (2011). How do patient characteristics influence informal payments for inpatient and outpatient health care in Albania: results of logit and OLS models using Albanian LSMS 2005. BMC Public Health, 11(1), 375. <https://doi.org/10.1186/1471-2458-11-375>
38. Toor IA, Butt MS. (2005). Determinants of health care expenditure in Pakistan. Pak Econ Soc Rev, 43(1), 133–150.
39. Uka A. (2014). Understanding informal patient payments in Kosovo's health care system health policy Institute; August 2014.
40. Vian T., Burak LJ. (2006). Beliefs about informal payments in Albania. Health Policy Plan, 21
41. Yetim, B., İlgün, G., Çilhoroz, Y., Demirci, Ş., & Konca, M. (2021). The socioeconomic determinants of health expenditure in OECD: An examination on panel data. International Journal of Healthcare Management, 14(4), 1265-1269.
42. Wagstaff, A. (2000). Socioeconomic inequalities in child mortality: comparisons across nine developing countries. Bulletin of the World Health Organization, 78, 19-29.
43. Wagstaff, A. (2002). Poverty and health sector inequalities. Bulletin of the world health organization, 80, 97-105
44. Wellalage, N. H., Fernandez, V., & Thrikawala, S. (2020). Corruption and innovation in private firms: Does gender matter?. International Review of Financial Analysis, 70, 101500. <https://doi.org/10.1016/j.irfa.2020.101500>
45. Were, V., Buff, A. M., Desai, M., Kariuki, S., Samuels, A., Ter Kuile, F. O., ... & Niessen, L. (2018). Socioeconomic health inequality in malaria indicators in rural western Kenya: evidence from a household malaria survey on burden and care-seeking behaviour. Malaria journal, 17, 1-10. <https://doi.org/10.1186/s12936-018-2319-0>
